# Unilateral Vocal Fold Paralysis and Voice Therapy: Predictors of Long-Term Quality of Life

**DOI:** 10.7759/cureus.34078

**Published:** 2023-01-23

**Authors:** Francisco Sousa, Mariline Santos, Sara Azevedo, Ana Pinto, Susana Vaz Freitas, Miguel Coutinho, Cecília Almeida e Sousa, Álvaro Moreira da Silva

**Affiliations:** 1 Otolaryngology - Head and Neck Surgery, Centro Hospitalar Universitário do Porto, Porto, PRT; 2 Intensive Care Unit, Centro Hospitalar Universitário do Porto, Porto, PRT

**Keywords:** dysphagia, time, quality of life, vhi-30, voice handicap index, voice therapy, unilateral vocal fold paralysis

## Abstract

To date, little is known about the long-term predictors of quality of life (QoL) in unilateral vocal fold paralysis (UVFP). The main objective of this study was to evaluate the predictors of long-term QoL in UVFP patients submitted to voice therapy (VT) exclusively. Data from patients diagnosed with UVFP who followed a VT program between 2013 and 2019 were reviewed. Video laryngoscopy (VL) records were obtained at the beginning and at the end of VT. To assess QoL, Voice Handicap Index 30 (VHI-30) score was assessed in three temporal frames: before voice therapy (pre-VT), at the last VT session (post-VT), and in the present (cur-VHI). A longitudinal analysis was performed regarding the evolution of QoL and the factors influencing QoL through time were analyzed. Seventy-eight percent of patients had iatrogenic UVFP. The mean time of follow-up after VT was 3.942 years (range 6 months-7 years). There was a significant improvement in QoL through all time points (F (2,88)=72.179, p<0.001), with VHI-30 decrease from the baseline pre-VT to post-VT(p<0.001) and from post-VT to cur-VT (p=0.0013). In the iatrogenic UVFP population, patients starting VT earlier showed better long-term QoL (p=0.023). UVFP patients with dysphagia at presentation showed significantly worse QoL in the late follow-up (p=0.016). Hence, iatrogenic UVFP patients beginning VT rapidly may show better QoL in the future. Also, our results suggest that dysphagia at UVFP onset may predict higher morbidity later in life.

## Introduction

Unilateral vocal fold paralysis (UVFP) is a frequent diagnosis in otolaryngology. The condition results in neural damage to recurrent laryngeal or superior laryngeal nerves and may present with dysphonia, dyspnea, and swallowing difficulty [1,2]. Causes such as cancer, trauma, and surgery are among the most frequently reported [1,2]. Laryngeal squamous cell carcinoma is the most common etiology of UVFP among neoplasms [3]. Inflammatory, infectious, and neurologic diseases are other potential causes. Idiopathic UVFP is an exclusion alternative when no other cause is found in the diagnostic workup, although some studies hypothesize an impending viral origin for this case [4-6].

UVFP can have a significant impact on a patient's quality of life (QoL). Hence, surgical and non-surgical interventions may be offered to achieve adequate vocal function. Surgical options such as temporary vocal fold augmentation and thyroplasty are some examples [7,8]. Voice therapy (VT) arises as a non-surgical approach by improving stabilization and elevation of the larynx while enhancing intrinsic muscle strength, glottal adduction, and supraglottic sphincters function and coordination [9,10].

There is insufficient data about the ideal timing for voice and/or swallowing therapy following iatrogenic injuries such as thyroidectomy [11]. Results from studies support the effectiveness of early VT in UVFP patients’ self-perception of vocal function as well as early motility recovery [7,12]. Studies do point to the importance of early VT programs in speech restoration in UVFP [1,7,13]. It is nevertheless obscure if the time taken between the first symptomology and first vocal therapy session impacts the prognosis in iatrogenic UVFP, and no guiding cutoff is given to referral timing. Consequently, it is here hypothesized that time to VT initiation may influence vocal outcomes longitudinally.

Regarding the evolution after VT cessation, it is unknown if there is a tendency for improvement in the vocal quality several months or years after the end of VT. Hence, this work aimed to assess the evolution of vocal handicap in the time following VT program termination. 

Much of the research on UVFP have focused on its implication on voice, as dysphonia constitutes the most common presenting complaint. Other symptoms include dysphagia, poor cough production, dyspnea, and stridor [14-16] and fewer data exist on its impact on patients' QoL. Studies refer to an incidence of about 60% of swallowing difficulty in UVFP patients and 75% of new-onset dyspnea [16-18]. Vocal fatigue, globus sensation, and neck discomfort are other subjective symptoms that may be associated with UVFP [7,10]. Only a few studies have specifically investigated the use of VT for treating dysphonia and dysphagia in patients who suffer from UVFP [11,19]. Therefore, we also evaluate whether accompanying presenting symptoms, namely dysphagia and dyspnea, can influence the outcome after VT. We hypothesize that dysphagia/dyspnea at presentation could predict poorer vocal QoL outcomes later in life. Finally, this study also evaluated whether UVFP etiology could itself predict future voice-related outcomes.

To summarize, the defined objectives of the study were 1) to assess if there is an improvement through time in voice-related QoL after the VT program; 2) to assess if time-to-VT initiation influences the voice outcomes longitudinally; 3) to assess if dysphagia/dyspnea at presentation impacts voice outcomes later in life; and 4) to assess if UVFP etiology relates to UVFP voice outcomes in the future.

## Materials and methods

Sample enrollment and evaluation

To perform a longitudinal analysis, data from patients diagnosed with UVFP who underwent VT as the sole treatment at the Department of Otolaryngology, Head and Neck surgery between 2013 and 2019 were reviewed. Patients suffering from other causes of dysphonia besides UVFP, or formerly submitted to laryngeal surgery or previous VT sessions were excluded. Patients ≥18 years, who were enrolled in a VT program with 100% adherence, who accepted to participate in the study and in whom objective and subjective assessments were available were enrolled. All the patients were proposed to VT and did not undergo any additional surgical intervention after the VT sessions, either because of good functional outcomes after VT or because they refused additional therapies. Data regarding sex, age, etiology, dyspnea or dysphagia at presentation, concurrent comorbidities, video laryngoscopy, and Voice Handicap Index 30 (VHI-30) were collected.

Objective and subjective measurements

The objective measurements of glottal competence were affeered by video laryngoscopy. Video laryngoscopy was obtained prior to the initiation of and at the last VT session. The position of the vocal fold was initially categorized into intermediate (Ip) or paramedian (Pp) relative to the midline. After VT, the final glottal status was categorized into complete glottal compensation (CGC) or anteroposterior glottal gap (APGG). Subjective measurement of voice handicap was assessed by using the VHI-30 questionnaire [20]. VHI-30 is a self-assessment questionnaire validated for many languages and made up of 30 questions with the primary goal of perceiving the impact that current voice function has on a patient's QoL [20-22]. It is further divided into domains (functional, emotional, and physical). VHI-30 scores before VT (pre-VT) and at the last VT session (post-VT) were collected based on clinical records. The current VHI-30 in 2020 (cur-VT) was assessed by telephone call. The time between the first vocal symptoms and the beginning of VT (Ti-to-VT), the total duration of VT sessions (VT-d), and the time from the last VT session to the current phone call (Ti-aft-VT) were also assessed.

Patients followed a VT protocol developed by Vaz-Freitas and Pedro Melo Pestana [10] and carried out by the same experienced speech and language pathologist. The patient was discharged from VT only when achieving an adequate functional ability or after >20 sessions without significant results. Patients who did not attend all the proposed sessions were excluded from the study. A convenient cutoff of 50 days was used for creating “early” (Ti-to-VT [<50]) vs “delayed” (Ti-to-VT [≥50]) treatment subgroups within the iatrogenic population - based on the median value of Ti-to-VT observed in that population.

Ethics

Informed consent was obtained for all patients. The study was approved by the Institutional Review Board and Ethics Committee of Centro Hospitalar Universitário do Porto (Number: 031-DEFI/032-CE) and the design complies with the Declaration of Helsinki ethical standards.

Statistical analysis

Statistical analysis was performed using SPSS (IBM SPSS Statistics Version 26.0; IBM Corp, Armonk, NY). In the descriptive analysis, categorical variables are presented as percentages, and continuous variables as means and standard deviations, or medians and interquartile range for variables with skewed distributions. Normal distribution was checked using skewness and kurtosis. The bivariate associations were analyzed using either an independent t-test (parametric analysis) or Mann-Whitney test (non-parametric analysis) depending on the tests for normality, Pearson's chi-square/Fisher's tests (95% confidence intervals) for categories and Spearman's test for continuous variables. A paired-samples t-test was also used to compare VHI-30 values in different time settings. To adjust for potential confounders, general linear models taking cur-VHI as the outcome were performed. A repeated-measures analysis of variance (ANOVA) was also performed. All reported p-values are two-tailed, with a p-value ≤0.05 indicating statistical significance.

## Results

Study population

The study population included a total of 59 Caucasian patients who underwent VT as the sole approach for UVFP. Forty-six (78%) were female and 13 (22%) were male. The mean age at the beginning of VT was 60.97 ± 12.42 years (range: 26-83 years). Thirty patients (50.8%) had left UVFP paralysis and 29 (49.2%) had right UVFP. Concerning etiology, 46 patients (78%) had iatrogenic UVFP (41 after thyroidectomy, two after parathyroidectomy, two after cervical spine surgery, and one after upper digestive endoscopy); 13 patients had non-iatrogenic UVFP: 10 patients (16.9%) had idiopathic UVFP, one patient infectious UVFP (1.7%), one patient neurogenic UVFP (1.7%), and one patient neoplastic UVFP (1.7%). The mean time after VT (Ti-aft-VT) was 3.942 ± 2.313 years (range 6 months-7 years). Before VT, 40 patients (68%) had UVFP in the intermediary position (Ip) and 19 patients (32%) had UVFP in the paramedian position (Pp). At the end of VT, 45 patients (76%) had CGC while 14 (24%) showed a residual APGG. Characteristics from other collected variables in our study's population are shown in Tables 1, 2.

**Table 1:** Descriptive analysis of categorical variables. ^1^p-Value refers to results from bivariate analysis comparison between iatrogenic and non-iatrogenic groups for each variable, using chi-square or Fisher's exact tests. Bold p values are statistically significant.

Categorical variables	Frequency (%)		p-Value^1^
	Iatrogenic	Non-iatrogenic	
Gender (male)	15.2	46.2	0.027
Presentation			
Dysphonia	100	93	0.775
Dysphagia	63	53,8	0.548
Dyspnea	54.3	38.5	0.312
Current dysphagia 2020	39.5	50	0.516
Time-to-voice therapy			
<50 days (Ti-to-VT [<50])	60.9	30.8	0.054
≥50 days (Ti-to-VT [≥50])	39.1	69.2	0.054
Comorbidities			
Smoking	24.4	7.7	0.264
Diabetes	15.6	23.1	0.678
Dyslipidemia	20.5	7.7	0.426
Hypertension	20	23.1	0.809
Laryngeal reflux	8.9	15.4	0.608
Neoplasia	28.9	7.7	0.159
Neurologic	4.4	23.1	0.069
Cardiac	13.3	15.4	0.850
Pulmonary	11.1	7.7	0.721
Auto-immune	6.7	7.7	0.540
Immunosuppression	4.4	7.7	0.898
Depression	26.7	0	0.051

**Table 2:** Descriptive analysis of registered continuous variables. SD, standard deviation; IQR, interquartile range. ^1^p-Value refers to results from bivariate analysis comparison between iatrogenic and non-iatrogenic groups for each variable, using Mann-Whitney test for medians and independent t-test for means. Bold p-values are statistically significant. ^2^Time between last VT session and 2020 phone call. *Impossible to measure accurately due to limitations in clinical records.

Continuous variables	Mean/median (SD, IQR)		p-Value^1^
	Iatrogenic	Non-iatrogenic	
Age at voice therapy (years)	60.52±11.66 SD	62.54±15.26 SD	0.665
Voice therapy duration (days) (VT-d)	77 (48-106)	56 (36.5-75,5)	0.045
Time-to-voice therapy (days) (Ti-to-VT)	45.5 (33,5-57,5)	Not measured*	-
Time-after- voice therapy (years) (Ti-aft-VT)^2 ^	3.732±2.410 SD	4.727±1.794 SD	0.146
Pre-therapy VHI-30 (pre-VHI)	48.49±21.37 SD	49.54±23.94 SD	0.889
Post-therapy VHI-30 (post-VHI)	18.46±20.86 SD	28.83±26.52 SD	0.228
Current (2020) VHI-30 (cur-VHI)	13.24±14.80 SD	20.00±23.10 SD	0.208
Functional	2.48±4.34 SD	6.10±8.10 SD	0.037
Emotional	2.80±5,02 SD	4.10±6.93 SD	0.545
Physical	7.93±7.41 SD	9.85±9.41 SD	0.509

Objective and subjective measurements: longitudinal assessment

Regarding video laryngoscopy, there was not a significant correlation between the position of the vocal fold prior to VT (Ip vs Pp) and the final glottal status after VT (CGC vs APGG) in the chi-square test (p=0.327).

VHI-30 decreased significantly from baseline (pre-VHI: 49±21) to the last VT session (post-VHI: 21±22), p<0.001. Also, the cur-VHI score mean (15±17) revealed a significant decrease compared to post-VHI (21±22), p=0.013. Results from repeated-measures ANOVA determined that VHI-30 scores differed significantly across the three time points (pre-VHI, post-VHI, and cur-VHI - F (2,88)=72.179, p<0.001).

Concerning the relationship between objective and subjective measures, the pre-VT vocal fold position did not significantly associate with pre-VHI (Ip: 50±21 vs Pp: 48±20, p=0.758), post-VHI (Ip: 20±22 vs Pp: 22±22, p=0.762) and cur-VHI (Ip: 14±13 vs Pp: 18±21, p=0.319). The post-VT glottal competence did not associate with pre-VHI (CGG: 47±22 vs APGG: 54±16, p=0.325), but did influence post-VHI (CGD: 15±20 vs APGG: 38±2, p=0.002). Post-VT glottal competence did not reach a significant association with cur-VHI although there was a tendency for significance (CGD: 13±16 vs APGG: 21±20, p=0.189).

Another objective was to investigate the effect of early vs delayed VT in future VHI-30. In this topic, further analysis was restricted to patients with iatrogenic etiology in order to minimize bias induced by differences in subgroups, limited sample size, and lack of precisive records on Ti-to-VT in the non-iatrogenic group. Hence, an analysis was performed to evaluate the association of Ti-to-VT and VHI-30 across different time frames. cur-VHI scores increased with longer Ti-to-VT values (Spearman's test, p<0.001). After adjusting for age (presbylarynx interference), pre-VT vocal fold position (Ip vs Pp), pre-VHI and VT duration (VT-d) in linear regression, the relation persisted, so that higher Ti-to-VT significantly associated with higher cur-VHI (see Table 3). Post-VT glottal competence did not associate with Ti-to-VT (CGD: 59±46 days vs APGG: 45±6 days, p=0.379).

**Table 3:** Results of regression model factors predicting the outcome “cur-VHI” adjusted for potential confounders. VHI-30, Vocal Handicap Index-30. ^1^β stands for unstandardized regression coefficient. ^2^Standard error for unstandardized regression coefficient. ^3^Standardized regression coefficient. ^4^t=β/SEβ. Potential confounders included in the regression: age (presbylarynx interference), vocal fold position at first assessment, baseline VHI and voice therapy duration.

Independent variable predicting current VHI-30	β^1^	SE β^2^	Beta^3^	t^4^	p-Value
Time-to-voice therapy onset (Ti-to-VT)	0.127	0.054	0.353	2.364	0.023
Time-to-voice therapy ≥ 50 days (Ti-to-VT [≥50])	14.307	4.171	0.477	3.430	0.001
Dysphagia at presentation	10.297	4.142	0.298	2.486	0.016

When accounted as categorical variables, the Ti-to-VT [≥50] group showed higher Cur-VHI mean values (independent-samples t-test p<0.001). Cur-VHI was also significantly higher for functional (p=0.001), emotional (p<0.001), and physical (p=0.003) domains in the Ti-to-VT [≥50] group. After adjustment for age (presbylarynx interference), pre-VT vocal fold position, pre-VHI, and VT-d, the relation persisted, with the Ti-to-VT [≥50] group showing higher cur-VHI (as shown in Table 3).

**Figure 1:**
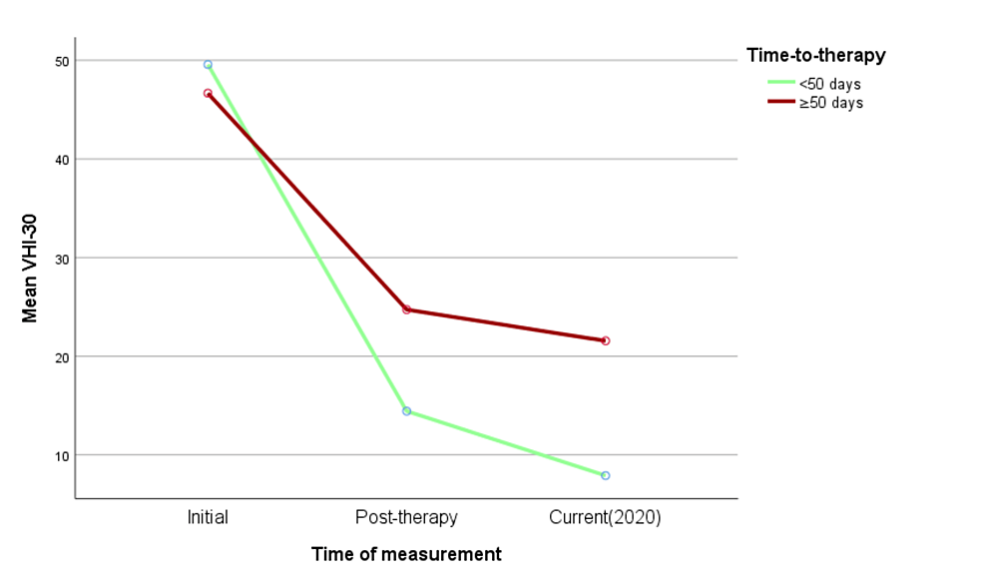
Mean VHI-30 score through different time assessments. Vocal Handicap Index 30 (VHI-30) values are similar before voice therapy. The slope reduction is higher across time in the early voice therapy group (<50 days). Note the significant improvement in the years that follow the end of sessions.

The initial pre-VHI did not differ between groups (p=0.638) (Figure 1). Results from ANOVA confirmed a significant effect of earlier VT in the long-term outcomes: cur-VHI proved to be significantly lower across time in the early Ti-to-VT [<50] group (F (2,88)=4.249, p=0.017). General linear model results for VHI-30 values across time and Ti-to-VT subgroups are shown in Figure 1. 

Clinical presentation

To evaluate the association between inaugural symptoms and future vocal outcomes, pre-VT focal fold position, post-VT glottal competence, and VHI-30 were compared between subgroups with and without dysphagia/dyspnea at UVFP onset.

Presenting with dysphagia did not associate with pre-VT vocal fold position (p=0.92) and post-VT glottal competence (p=0.774). Likewise, presenting with dyspnea did not associate with pre-VT focal fold position (p=0.86) and post-VT glottal competence (p=0.056).

Presenting with dysphagia associated with higher cur-VHI (p=0.016) and even more with cur-VHI physical score (p=0.002). In the multivariate analysis with adjustment for age, pre-VT focal fold position, pre-VHI, and VT-d, the statistically significant relationship between dysphagia at presentation and higher cur-VHI persisted (see Table 3). Also, dysphagia at UVFP presentation significantly correlated with current (2020) dysphagia reports (p<0.001).

An association between presenting with dyspnea and cur-VHI was not found (p=0.395). Likewise, no association was found between initial dyspnea and functional (p=0.968), emotional (p=0.908), or physical (p=0.082) cur-VHI index. No associations were seen between dyspnea at presentation and any comorbidity (p>0.05). Presenting with dyspnea did not associate with the presence of current (2020) dysphagia (p=0.874).

Etiology

To assess whether etiology influences the long-term prognosis of UVFP, pre-VT vocal fold position, post-VT glottal competence, and VHI-30 were compared between iatrogenic and non-iatrogenic groups through time.

Pre-VT vocal fold position did not associate with UVFP etiology (p=0.371). Pre-VHI (p=0.889), post-VHI (p=0.228), and cur-VHI (p=0.208) did not statistically differ between groups in the independent-samples t-test. When analyzed separately, the functional cur-VHI was higher in the non-iatrogenic group, but no differences were found in the emotional and physical values (see Table 2).

The post-VHI significantly decreased in both iatrogenic (paired-samples t-test, p<0.001) and non-iatrogenic patients (paired-samples t-test, p=0.015).

An additional comparative analysis was performed regarding other clinical and demographic characteristics, as shown in Table 1. A significant difference was found between iatrogenic and non-iatrogenic groups regarding sex since females had more than a four-fold increased risk for iatrogenic etiology (Fisher's exact test p=0.027; odds ratio 4.775). There was not a significant difference between the two groups concerning age, comorbidities, presenting symptoms, or current (2020) dysphagia (see Table 1). VT-d was significantly higher in the iatrogenic group compared to the non-iatrogenic group (Table 2).

## Discussion

One of the objectives of this study was to evaluate the long-term QoL in UVFP after leaving VT sessions. Long-term results of VT are generally lacking in the literature. Little is known about whether there is a tendency for improvement in the vocal handicap several months or years after the end of VT in UVFP. Does time itself influence the process of individual adaption to this disease? Should one expect an additional improvement in VHI-30 many months/years after the cessation of the VT program? To explore this topic, the evolution of VHI-30 through time was assessed. Our results showed significant improvement in the VHI-30 through time that persisted after cessation of VT (Figure 1), irrespective of vocal fold position and glottal competence. These findings may translate the effect of time and personal adaptation on functional recovery after VT [12,23]. Other studies that included a UVFP group not submitted to VT or any other intervention also showed improvement in phonation and videoendoscopic reductions in glottal gap size with time [12]. Similarly, in our study, there was a significant improvement in vocal self-reports months/years after the completion of VT. Possible explanations may include vocal recovery in the absence of restored vocal fold motion, but also recruitment of residual innervations/reinnervation with motion gain [12,24].

On the other hand, studies concerning Ti-to-VT and voice handicaps are still scarce, although some point to early VT as an important determinant of vocal improvement in the future [12,19,25-27]. No consensus seems to exist on cutoff timings for VT referral [12,19,25,26]. Literature supports the effectiveness of early VT in UVFP patients’ self-perception of vocal function as well as early motility recovery, suggesting the efficacy of early VT programs in speech restoration [1,7-9,12,13,19,26]. There is also some evidence that delayed VT can have negative impacts on voice outcomes [28]. Nevertheless, it is still a matter of debate if the time taken between the first symptoms and the first VT session influences the long-term prognosis in iatrogenic UVFP [19]. Thus, we also studied the impact of Ti-to-VT on long-term VHI-30 in iatrogenic UVFP patients. The results here presented suggest that patients suffering an iatrogenic injury who initiate VT earlier have better VHI-30 in the long term, irrespective of post-VT glottal competence. The cur-VHI was significantly lower in the Ti-to-VT [<50 days] group, pointing to a beneficial effect of early VT extending through time. These findings, if highly replicated, could stimulate the development of well-defined coordination strategies, so that patients with iatrogenic injuries could begin VT promptly. Although one could theorize that better outcomes in the early VT group could be in part related to the prompt reduction of the glottal gap, our results do not favor this hypothesis, since no association was found between post-VT glottal competence and time to VT initiation. Thus, we alternatively theorize that the beneficial effect of early VT can be explained by a prompt determent in the development of noxious compensatory behaviors [9,12].

Regarding presenting symptoms, dysphagia proved to be a significant prognostic marker of poorer vocal function in UVFP. There could be a theoretical rationale to associate dysphagia and voice handicap in these patients: in UVFP, dysphagia relates to glottal incomplete closure, since maintenance of the closed glottis is essential to raise the subglottic pressure for an efficient swallow [29-32]. Likewise, good voice production also depends on glottal closure, so the farther the paralyzed cord rests toward the midline the lower the chance for adequate phonation [17]. Nevertheless, in our sample, dysphagia at UVFP onset did not relate neither with pre-VT vocal fold position (Ip vs Pp) nor with post-VT glottal competence (CGC vs APGG). Therefore, the basis for these results may not rely directly on endoscopic findings but on other underlying mechanisms such as laryngeal sensitivity. Dyspnea at presentation, on the other hand, did not relate to VHI-30 severity in the future. Similarly, Brunner et al. did not find a significant correlation between subjective breathing impairment and the position of the paralyzed vocal fold or glottic width [17]. Dyspnea in UVFP might be alternatively explained by neurosensorial deficits of the laryngeal mucosa or by the discrepancy between effort and output of breathing [17]. 

A secondary objective was to compare UVFP subgroups concerning objective and subjective outcomes through time, in order to analyze if UVFP etiology could affect perceived vocal handicap later in life. The pre-VT vocal fold position, post-VT glottal competence, post-VHI, and cur-VHI did not differ between groups; hence, the etiology per se did not seem to predict vocal handicap. Significant improvements existed in perceived QoL measured by VHI-30 score at the end and long after VT cessation in all groups, irrespective of etiology. Iatrogenic etiology was more prevalent in females possibly due to a higher incidence of thyroid surgery indication [33].

The present study has limitations. VHI-30 is itself a subjective measurement, which evaluates QoL, thus depending on the patient's experience of the disease. One of the major limitations of the study is the lack of precise timing for non-iatrogenic lesions, which made the measurement of time-to-VT initiation impossible to calculate in this subpopulation. The sample size is relatively limited due to the strict eligibility criteria. Also, the last VHI-30 assessment was also performed by a telephone call which could incur in bias. 

This study has its own strengths as well. It is the first to show a significant improvement in VHI-30 years after cessation of VT. Importantly, it is also the first study to demark dysphagia at onset as a poor vocal prognostic indicator in UVFP. It also raises awareness for the benefit of early VT enrollment in UVFP, suggesting better long-term outcomes when enrollment is done less than 50 days from onset. 

Further research with a larger number of patients, a control group not undergoing voice therapy or undergoing other rehabilitation methods, the use of diverse vocal function instruments, and including a larger non-iatrogenic UVFP sample would be pertinent. 

## Conclusions

This study supports that an improvement in vocal function may occur long after the end of VT. Iatrogenic UVFP patients initiating VT earlier had better long-term VHI-30. Thus, time to VT onset seems to impact the future perceived vocal handicap in iatrogenic UVFP. The prognostic benefits of an early VT program enrolment suggest the importance of a timely referral to VT for those patients. Dysphagia at onset may predict long-term aggravated vocal handicap.
